# Investigation of potential traces of pluripotency in germinal-center-derived B-cell lymphomas driven by *MYC*

**DOI:** 10.1038/bcj.2015.40

**Published:** 2015-05-29

**Authors:** R Wagener, M Lenz, B Schuldt, I Lenz, A Schuppert, R Siebert, F-J Müller

**Affiliations:** 1 Institute of Human Genetics, Christian-Albrechts-University Kiel and University Hospital Schleswig-Holstein, Kiel, Germany; 2 Institute for Advanced Study in Computational Engineering Science (AICES), RWTH Aachen University, Aachen, Germany; 3 Joint Research Center for Computational Biomedicine, RWTH Aachen University, Aachen, Germany; 4 Zentrum für Integrative Psychiatrie, University Hospital Schleswig-Holstein, Kiel, Germany

Human pluripotent stem cells (hPSCs) are defined by their indefinite self-renewal *in vitro* and their potential to differentiate into cells representative of all lineages of the mammalian gastrulation embryo.^1^ Several similarities link these genomically normal cells with features salient to malignancies,^2^ for example, when injected into immunodeficient mice, hPSCs give rise to tumors closely resembling teratomas.^1^ hPSCs can be induced from somatic cells by the transient overexpression of four genes (*POU5F1, KLF4, SOX2* and *MYC*). The induction of pluripotency in somatic cells shares again several molecular mechanisms with malignant transformation. Moreover, all of those four reprogramming factors possess oncogenic potential,^2^ with *MYC* being the most prominent oncogene activated in a wide range of malignancies. Among them, the prototype of a *MYC*-driven tumor is Burkitt lymphoma (BL).

BL is the most common B-cell lymphoma in childhood. Its biological hallmark are translocations juxtaposing the *MYC* oncogene next to one of the three immunoglobulin (IG) loci. Such an *IG–MYC* fusion resulting in a deregulated MYC expression is present in nearly all BL.^3^ In contrast to other B-cell lymphomas, in which *IG–MYC* or non-*IG–MYC* translocations frequently occur during clonal evolution and disease progression, it is widely accepted that the *IG–MYC* translocation is the initiating event in Burkitt lymphomagenesis.^4^

Studies in chicken bursa of Fabricius have shown that *myc* overexpression induces preneoplastic lesions, which subsequently lead to lymphoma in a cell population solely present in B-cell differentiation at the bursal stem cell stage.^5, 6^ Moreover, BL cells are exceptional among germinal-center-derived B-cell lymphomas, in that they usually show a dark zone gene expression profile and the dark zone is assumed to be the initiating part in the evolution of germinal centers after antigen stimulation.^7^ These findings as well as the incidence peak of BL between 4 and 7 years of age and the pluripotency-promoting capacity of *MYC* led us to the hypothesis that, BL might derive from or resemble a B-lymphoid cell with—potential—stem cell features.

To investigate whether BL cells share characteristics with hPSCs, we used a recently published bioinformatic assay to determine pluripotency in human cells.^8^ By this unbiased approach, we compared a highly discriminative, transcriptional model derived from a large database of hPSC gene expression profiles with genome-wide transcriptional profiles from various germinal center B-cell lymphoma entities including BL.

Gene expression data from BL, as well as other *MYC*-positive and *MYC*-negative mature aggressive B-cell lymphomas obtained by use of the Affymetrix (Santa Clara, CA, USA) U133A platform have been published previously.^9^ The raw dataset applied for the present analysis (GSE4475) included a total of 221 mature aggressive B-cell lymphomas classified as molecular BL (mBL, *n*=44), non-mBLs (*n*=129) and lymphomas with a gene expression signature intermediate between mBL and non-mBL (intermediate lymphomas, *n*=48). Moreover, the cases were classified according to their *MYC* translocation status into those having a translocation juxtaposing *MYC* with an *IG* locus (*IG–MYC* positive), with a non-*IG* locus (non-*IG–MYC* positive) or lacking a *MYC* translocation (*MYC* negative). For contextualizing our findings, raw gene expression data of 173 primary germ cell tumors (GCTs) (GSE3218, GSE10783 and GSE18155) and from a large expression atlas (E-MTAB-62 (ref. 10)) were downloaded. All raw data sets were preprocessed as described in the Supplementary Materials and Methods section.

Using the PluriTest algorithm, mBL could not be classified as pluripotent (Figure 1a). In comparison, most GCTs and teratomas showed strikingly higher Pluripotency Scores and lower Novelty Scores (Supplementary Figure S1), indicating a closer resemblance of most GCT transcription profiles to hPSC than any of the analyzed lymphomas to hPSCs.

Interestingly, when we compared the subgroups of the 221 analyzed mature aggressive B-cell lymphomas, we found that mBL scored slightly higher on the Pluripotency Score and lower on the Novelty Score than *MYC*-negative non-mBL (Figure 1b), effectively separating those two tumor entities in two groups (*P*-value<10^−17^) based on the resemblance to transcriptional patterns characteristic for hPSCs. To elucidate whether the translocation and subsequent overexpression of *MYC* leads to the increased Pluripotency Score and overall similarity to hPSCs as determined by the Novelty Score, non-mBL and intermediate B-cell lymphoma were color-coded based on their *MYC* translocation status (*IG–MYC*, non-*IG–MYC* or *MYC* negative). Figure 1c shows that the intermediates and non-mBL being *MYC*-positive mostly do not have a higher Pluripotency Score than the *MYC*-negative but are intermingled between mBL and *MYC*-negative non-mBL, suggesting that *MYC* status alone cannot explain the higher Pluripotency Score and lower Novelty Score of mBL compared with non-mBL.

Nevertheless, we find that in mBL 44.8% of Pluripotency Score signature genes are expressed at comparable levels to embryonic stem cells (Figure 1d). This is in line with recent findings that tumor cells share an overall gene expression pattern with PSC,^11^ which might be caused by the shared molecular and cellular features as, for example, certain genes drive a rapid proliferation rate both in mBL and hPSC.^1^ Along these lines, Kim *et al.*^12^ have shown that the similarities of hPSCs and some tumor-associated signatures are contributed by a distinct *MYC* regulatory network configuration. This agrees with our subsequent analyses, where we find that most mBL barely express genes mechanistically linked to pluripotency such as *NANOG* or *POU5F1* (refer to Figure 1d), but express genes that are related to increased cell cycle and proliferative activity as *BCL11A* or *PLCG2* at relatively high levels. Therefore, it is possible that both Pluripotency Score and Novelty Score are shifted toward hPSCs by a MYC-driven proliferation signature.

To summarize, using the PluriTest algorithm we could show that BL cells resemble more hPSCs than non-mBLs and intermediate lymphomas. In spite of this clear and distinguishing shift in the unbiased PluriTest Scores, we were not able to verify our hypothesis that BL might derive from or resemble cells with PSC features.

## Figures and Tables

**Figure 1:**
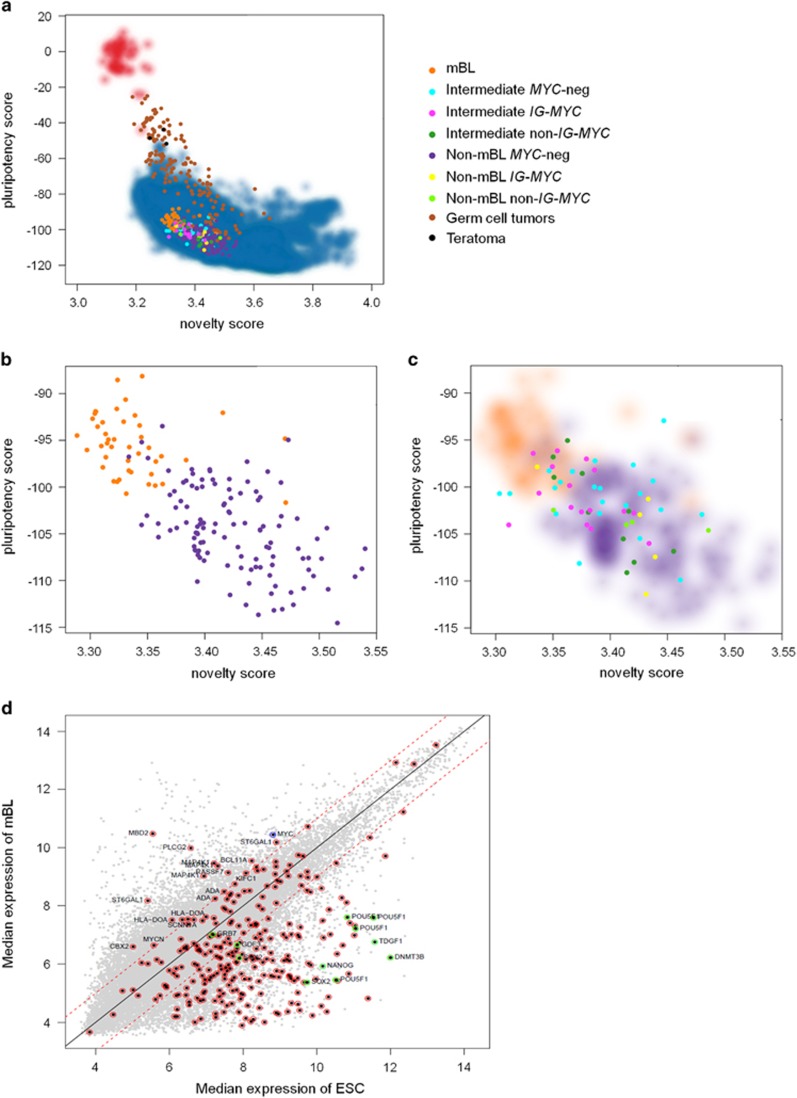
PluriTest analysis. (**a**) PluriTest results in germ cell tumors, somatic tissues and B-cell lymphoma. The red and blue background encodes an empirical density map indicating the location of pluripotent (red) and non-pluripotent (blue) cells in the reference data set. (**b**) Zoom-in of the PluriTest results, now only shown for mBL and *MYC*-negative non-mBL showing a visible separation between those two groups. (**c**) Using the mBL and *MYC*-negative non-mBL as an empirical density map, intermediate B-cell lymphomas and non-mBL, which were differentiated based on their *MYC* translocation status (*MYC*-neg, *IG–MYC* and non-*IG–MYC*), were integrated into the PluriTest. The latter lymphoma cases do not separate according to their *MYC* status in different groups. (**d**) Pluripotency plot comparing the median gene expression of 44 mBLs and 43 embryonic stem cells with special regard to the expression of pluripotency signature genes.^8^ Overall, 44.8% of the pluripotency signature genes are expressed at comparable levels in mBL as in embryonic stem cells. Thirteen of those genes are significantly upregulated in mBL, of which most can be attributed to genes associated with increased cell cycle and proliferative activity. The gray dots represent all genes that were analyzed on the gene array. Dots outlined in red mark genes of the pluripotency signature, dots outlined in green are genes linked to pluripotency and the blue dot depicts the *MYC* gene. The dashed red line denotes the log-fold change of ±1.
