# Supplementary figures and images for: Investigation of potential traces of pluripotency in germinal-center-derived B-cell lymphomas driven by MYC
Source: Blood Cancer J. 2015 May 29;5(5):e317–. doi: 10.1038/bcj.2015.40 (PMC4476019; doi:10.1038/bcj.2015.40)

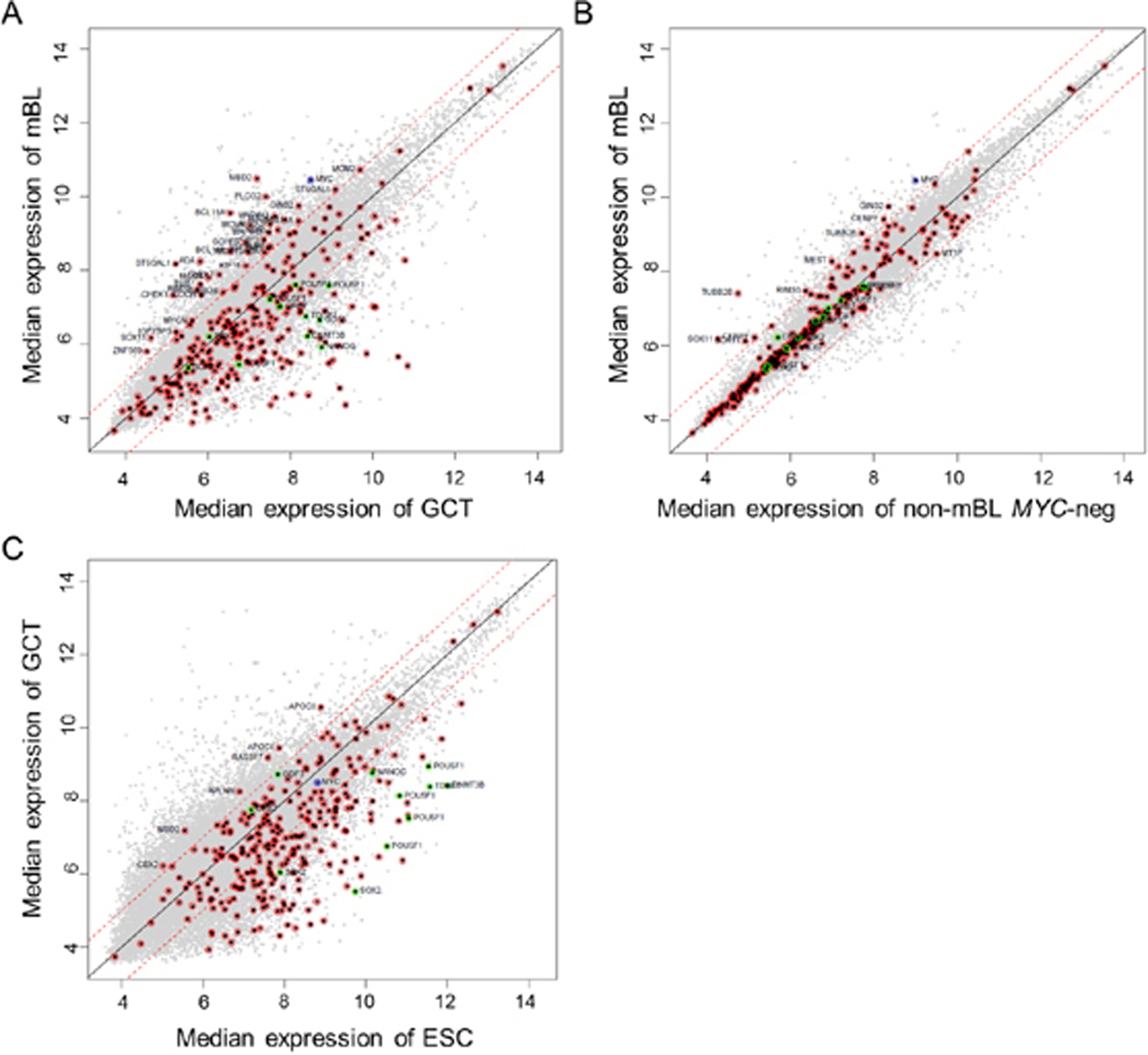

Supplement: Supplementary Figure S1 [file bcj201540x1.tif]
