# Supplementary material for: Investigation of potential traces of pluripotency in germinal-center-derived B-cell lymphomas driven by MYC
Source: Blood Cancer J. 2015 May 29;5(5):e317–. doi: 10.1038/bcj.2015.40 (PMC4476019; doi:10.1038/bcj.2015.40)
Supplement: Supplementary Information [file bcj201540x2.doc]

**LETTER TO THE EDITOR**

**Investigation of potential traces of pluripotency in germinal-center derived B-cell lymphomas driven by MYC**

Rabea Wagener^1^, Michael Lenz^2,3^, Bernhard Schuldt^2,3^, Insa Lenz^4^, Andreas Schuppert^2,3^, Reiner Siebert^1^, Franz-Josef Müller^4^

^1^Institute of Human Genetics, Christian-Albrechts-University Kiel & University Hospital Schleswig-Holstein, Campus Kiel, Kiel, Germany

^2^ Institute for Advanced Study in Computational Engineering Science (AICES), RWTH Aachen University, Aachen, Germany

^3^Joint Research Center for Computational Biomedicine, RWTH Aachen University, Aachen, Germany

^4^Zentrum für Integrative Psychiatrie, University Hospital Schleswig-Holstein, Campus Kiel, Kiel, Germany

**Supplementary Information**

**Material and Methods**

***Data preprocessing***

The raw lymphoma data (GSE4475 ^1^), germ cell tumor data (GSE3218 ^2^, GSE10783 ^3^, GSE18155 ^4^), and teratoma data (GSE13586 ^5^) were downloaded from Gene expression omnibus (http://www.ncbi.nlm.nih.gov/geo/) and RMA normalized with Affymetrix Power Tools.

The RMA preprocessed data from ^6^ were downloaded from ArrayExpress (accession number E-MTAB-62).

Due to the relatively small amount of embryonic stem cell data in the Lukk et al. ^6^ dataset, we additionally used the pluripotent stem cell data from GSE7332 ^7^, GSE25970 ^8^, and GSE47466 ^9^ for our analyses. These data were preprocessed in the same way as the lymphoma and germ cell tumor data. All data were quantile normalized together in order to reduce systematic study specific differences.

Differences between the Affymetrix Human Genome U133A and the HT Human Genome U133A arrays (study GSE25970 ^8^) were negligible for our rather global analysis after normalization.

***PluriTest Analysis, statistical testing, and gene selection***

The PluriTest bioinformatics assay was originally developed on the Illumina Human HT12 v3 microarray platform. For a transformation to the Affymetrix Human U133A platform, probes were mapped via the biomaRt package (version 2.20.0) in R (version 3.1.0).

Pluripotency and novelty scores were calculated as described before.^10^

The p-value between the mBL and non-mBL samples on the pluripotency and novelty scores was calculated using a Wilcoxon rank sum test. The exact p-values were 5.65*10^-18^ for the novelty score and 3.24*10^-19^ for the pluripotency score.

The pluripotency associated genes used in the scatter plot have been extracted as described in ^10^. Briefly, the algorithm of Kim *et* al. ^11^ as implemented in the extractFeatures method of the NMF package in R ^12^ was used to determine the genes that are most relevant for a pluripotency associated dimension in the NMF model.

***Reference dataset:***

| Dataset | Microarray platform | # Pluripotent | # Non-pluripotent |
| --- | --- | --- | --- |
| E-MTAB-62 | Affy HG-U133A | 6 | 5295 |
| GSE25970 | Affy HT_HG-U133A | 32 | 0 |
| GSE7332 | Affy HG-U133A | 3 | 0 |
| GSE47466 | Affy HG-U133A | 2 | 0 |

***Lymphoma, germ cell tumor, and teratoma data:***

| Dataset | Platform | # Lymphoma | # GCT | # Teratoma |
| --- | --- | --- | --- | --- |
| GSE4475 | Affy HG-U133A | 221 | 0 | 0 |
| GSE3218 | Affy HG-U133A | 0 | 101 | 0 |
| GSE10783 | Affy HG-U133A | 0 | 34 | 0 |
| GSE18155 | Affy HG-U133A | 0 | 38 | 0 |
| GSE13586 | Affy HG-U133A | 0 | 0 | 3 |

**References**

1 Hummel M, Bentink S, Berger H, Klapper W, Wessendorf S, Barth TFE *et al.* A biologic definition of Burkitt’s lymphoma from transcriptional and genomic profiling. *N Engl J Med* 2006; 354: 2419–2430.

2 Kushwaha R, Jagadish N, Kustagi M, Tomishima MJ, Mendiratta G, Bansal M *et al.* Interrogation of a context-specific transcription factor network identifies novel regulators of pluripotency. *Stem Cells Dayt Ohio* 2014. doi:10.1002/stem.1870.

3 Korkola JE, Houldsworth J, Feldman DR, Olshen AB, Qin L-X, Patil S *et al.* Identification and validation of a gene expression signature that predicts outcome in adult men with germ cell tumors. *J Clin Oncol Off J Am Soc Clin Oncol* 2009; 27: 5240–5247.

4 Palmer RD, Murray MJ, Saini HK, van Dongen S, Abreu-Goodger C, Muralidhar B *et al.* Malignant germ cell tumors display common microRNA profiles resulting in global changes in expression of messenger RNA targets. *Cancer Res* 2010; 70: 2911–2923.

5 Blum B, Bar-Nur O, Golan-Lev T, Benvenisty N. The anti-apoptotic gene survivin contributes to teratoma formation by human embryonic stem cells. *Nat Biotechnol* 2009; 27: 281–287.

6 Lukk M, Kapushesky M, Nikkilä J, Parkinson H, Goncalves A, Huber W *et al.* A global map of human gene expression. *Nat Biotechnol* 2010; 28: 322–324.

7 Barberi T, Bradbury M, Dincer Z, Panagiotakos G, Socci ND, Studer L. Derivation of engraftable skeletal myoblasts from human embryonic stem cells. *Nat Med* 2007; 13: 642–648.

8 Bock C, Kiskinis E, Verstappen G, Gu H, Boulting G, Smith ZD *et al.* Reference Maps of human ES and iPS cell variation enable high-throughput characterization of pluripotent cell lines. *Cell* 2011; 144: 439–452.

9 Lee JB, Werbowetski-Ogilvie TE, Lee J-H, McIntyre BAS, Schnerch A, Hong S-H *et al.* Notch-HES1 signaling axis controls hemato-endothelial fate decisions of human embryonic and induced pluripotent stem cells. *Blood* 2013; 122: 1162–1173.

10 Müller F-J, Schuldt BM, Williams R, Mason D, Altun G, Papapetrou EP *et al.* A bioinformatic assay for pluripotency in human cells. *Nat Methods* 2011; 8: 315–317.

11 Kim H, Park H. Sparse non-negative matrix factorizations via alternating non-negativity-constrained least squares for microarray data analysis. *Bioinforma Oxf Engl* 2007; 23: 1495–1502.

12 Gaujoux R, Seoighe C. A flexible R package for nonnegative matrix factorization. *BMC Bioinformatics* 2010; 11: 367.

**Supplementary Figure Legend**

**Figure SI.** Pluripotency Plot comparing the median gene expression of mBL with GCT (A), mBL with MYC-negative non-BL (B) and GCT with ESC (C) with special regard to the expression of pluripotency signature genes.^10^ The grey dots represent all genes which were analyzed on the gene array. Dots outlined in red mark genes of the pluripotency signature, dots outlined in green are genes linked to pluripotency and the blue dot depicts the *MYC* gene. The dashed red line denotes the log-fold change of ±1.
